# Unusual Hypermucoviscous Clinical Isolate of Klebsiella pneumoniae with No Known Determinants of Hypermucoviscosity

**DOI:** 10.1128/spectrum.00393-22

**Published:** 2022-06-01

**Authors:** Tamal Dey, Ardhendu Chakrabortty, Aastha Kapoor, Anuja Warrier, Vijaya Lakshmi Nag, Karthikeyan Sivashanmugam, Manoharan Shankar

**Affiliations:** a Department of Bioscience and Bioengineering, Indian Institute of Technology Jodhpur, Jodhpur, Rajasthan, India; b Department of Microbiology, All India Institute of Medical Sciences Jodhpur, Jodhpur, Rajasthan, India; c School of Bio Sciences and Technology, Vellore Institute of Technology University, Vellore, Tamil Nadu, India; Institut Pasteur

**Keywords:** *Klebsiella pneumoniae*, hypermucoviscosity, capsule, capsular polysaccharide, virulence

## Abstract

Klebsiella pneumoniae can be broadly classified into classical strains that cause drug-resistant, hospital-associated infections and hypervirulent strains that cause invasive, community-acquired, drug-susceptible infections. Hypermucoviscosity in Klebsiella pneumoniae has been associated with immune evasion and hypervirulence. A string-test-positive, hypermucoviscous strain of Klebsiella pneumoniae, P34, was isolated from the cystic lesion of a patient who reported to a tertiary care hospital in Jodhpur, Rajasthan, India. Given the antibiotic-susceptible and hypermucoviscous nature of the isolate, it was suspected to belong to the hypervirulent lineage of Klebsiella pneumoniae. However, P34 did not overproduce capsular polysaccharides and also remained susceptible to the antimicrobial effects of human serum when tested alongside strains that were non-hypermucoviscous. Sequencing of the genome of P34 revealed the absence of any large virulence plasmids or integrative conjugative elements that usually carry hypermucoviscosity- and hypervirulence-associated genes. P34 also lacked key virulence determinants such as aerobactin, yersiniabactin, and salmochelin biosynthesis clusters. In addition, P34 lacked homologs for genes associated with enhanced capsule synthesis and hypermucoviscosity, such as *rmpA*, *rmpA2*, *rmpC*, and *rmpD* (regulator of mucoid phenotype). These observations suggest that P34 may harbor novel genetic determinants of hypermucoviscosity independent of the indirectly acting *rmpA* and the recently described *rmpD*.

**IMPORTANCE** Hypermucoviscosity is a characteristic of hypervirulent Klebsiella pneumoniae strains, which are capable of causing invasive disease in community settings. This study reports phenotyping and genomic analysis of an unusual clinical isolate of Klebsiella pneumoniae, P34, which exhibits hypermucoviscosity and yet does not harbor *rmp* (regulator of mucoid phenotype) genes, which are known determinants of hypermucoviscosity (*rmpA* and *rmpD*). Similar clinical isolates belonging to the K. pneumoniae complex that are hypermucoviscous but do not harbor the *rmp* loci have been reported from India and abroad, indicating the prevalence of unknown determinants contributing to hypermucoviscosity. Therefore, strains like P34 will serve as model systems to mechanistically study potentially novel determinants of hypermucoviscosity in the K. pneumoniae complex.

## OBSERVATION

Classical Klebsiella pneumoniae strains usually cause nosocomial infections that are difficult to treat in immunocompromised individuals due to high levels of drug resistance (1). Newer, hypervirulent strains of K. pneumoniae with acquired virulence factors that can cause invasive infections (e.g., pyogenic liver abscesses and cholecystitis) in immunocompetent individuals in community settings have also emerged (2). Both lineages independently and their potential for convergence present serious threats to public health (3) and have led to K. pneumoniae being considered a priority pathogen. Several hypervirulent strains tend to be hypermucoviscous and also produce copious amounts of specific capsular polysaccharides (CPS). Although production of CPS in K. pneumoniae has been investigated extensively, the genetic basis of hypermucoviscosity (HMV) has not been interrogated systematically until recently. Earlier, HMV and CPS overproduction were thought to be overlapping traits because loss of the *rmpA* locus resulted in loss of HMV and caused a reduction in CPS production (4). This led to the incorrect association of the *rmpA* locus with the HMV phenotype, although the mechanism was unknown. A recent study showed that RmpA activates expression of a transcriptional unit including *rmpA*, *rmpD*, and *rmpC*. The loss of the HMV phenotype upon inactivation of *rmpA* was shown to be due to reduced expression of *rmpD*, while reduction in capsule synthesis was shown to be due to reduced expression of *rmpC* (5). The observation that the loss of *rmpC* results in lower CPS production but does not have any effect on the HMV phenotype (6) and that *rmpD* encodes a small membrane protein that causes HMV in K. pneumoniae KPPR1S with no influence on capsule production (5) indicated that HMV and CPS production were separable traits. However, it should be noted that some elements of CPS production are necessary for HMV, as strains with mutations in CPS biosynthesis genes (*manC* and *wcaJ*) also lose HMV irrespective of whether *rmpD* is functional (5).

**FIG 1:**
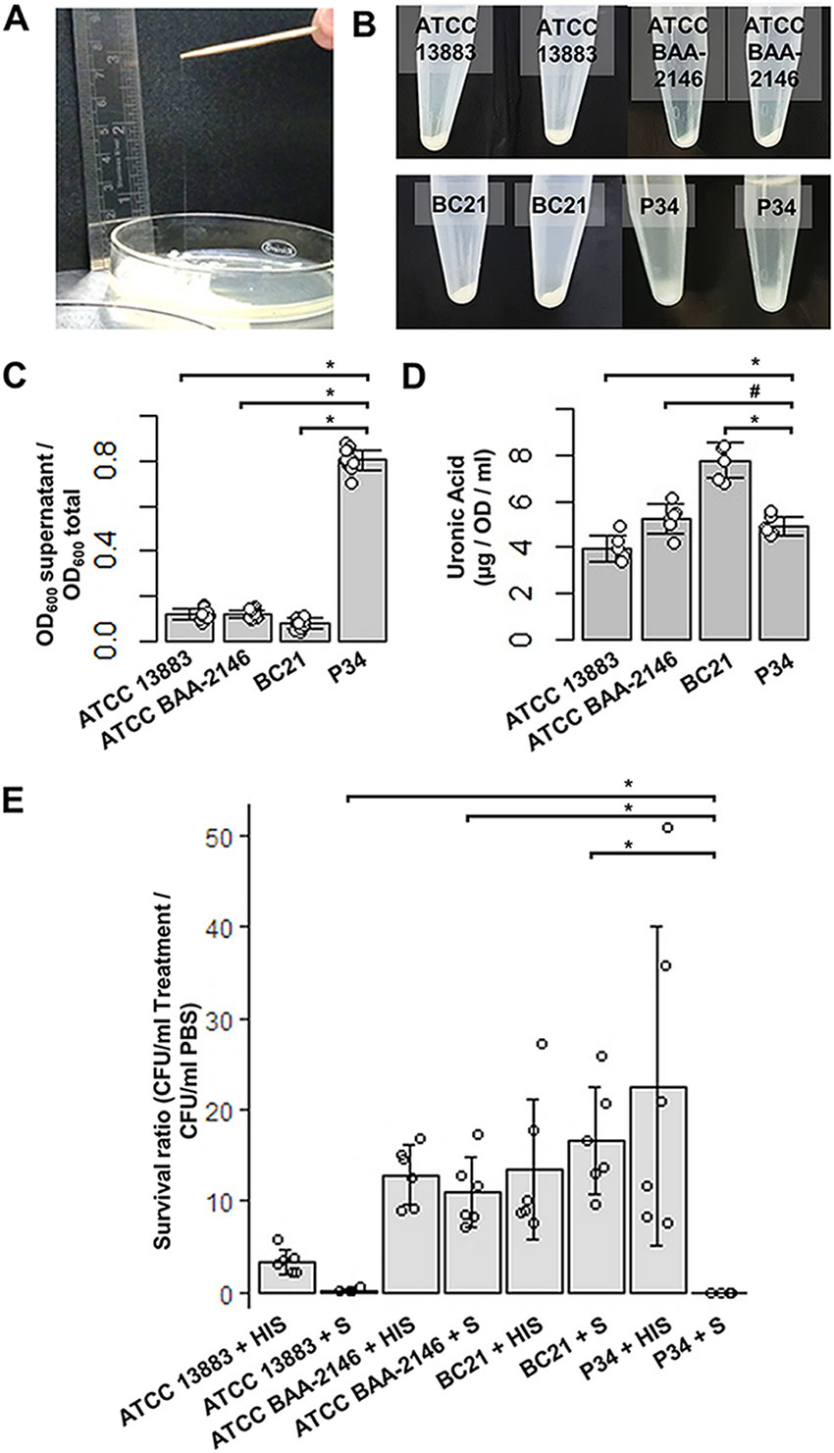
Phenotyping of Klebsiella pneumoniae P34. (A) String test, showing a string of ~65 mm produced when a single colony of P34 was touched with a wooden applicator, which was then moved away from the surface of the colony. A string with a length of >5 mm is usually considered positive. (B) Sedimentation and pellet formation by suspensions of P34 and reference strains in a low-speed centrifugation (1,000 × *g* for 5 min) assay (8). P34 sedimented poorly and formed extremely loose pellets, while all other strains tested formed tight pellets (C) Quantification of the hypermucoviscosity of P34 using the sedimentation assay (8). P34 and the reference strains were suspended in PBS and centrifuged at 1,000 × *g* for 5 min, after which the OD_600_ supernatant/total ratio was calculated. Each point corresponds to an independent biological replicate (*n* = 13). Bars indicate the mean ± standard deviation for all measurements. Assays were performed in five independent temporal batches, with 2 or 3 biological replicates per batch. (D) Amounts of CPS produced by P34 and reference strains as measured by the uronic acid assay (6). Each point corresponds to an independent biological replicate (*n* = 6). Bars show the mean ± standard deviation for all measurements. Assays were performed in three independent temporal batches, with 2 biological replicates per batch. (E) Susceptibility of P34 and reference strains to the antimicrobial effects of human serum, expressed as survival ratios (number of viable cells per milliliter after exposure to serum or heat-inactivated serum for 2 h divided by the number of viable cells per milliliter after exposure to PBS for the same time) (9). S, serum treatment; HIS, treatment with heat-inactivated serum. Each point corresponds to an independent biological replicate (*n* = 6). Bars show the mean ± standard deviation for all measurements. Assays were performed in three independent temporal batches, with 2 biological replicates per batch. A mean survival ratio of <1 indicated sensitivity to human serum, while one of >1 indicated resistance. All measurements (capsule production, hypermucoviscosity, and serum survival ratios) for P34 were compared with those for the other strains tested, using a Welch two-sample *t* test. *, *P* values of <0.05 were considered significant; #, *P* values of >0.05 were insignificant.

P34 was isolated from a 67-year-old female patient who was brought to a tertiary care hospital in Jodhpur, India, in August 2020 with complaints of abdominal pain, constipation, and anorexia for about 3 to 4 weeks. P34 was isolated from the pus aspirated from the cystic component of a solid cystic lesion involving the fundus and medial wall of the body of the gallbladder in the infrahepatic region. It may be noteworthy that the patient was a known case of smooth muscle neoplasm of the gallbladder and had defaulted treatment for 3 months. P34 was susceptible to most antibiotics tested (cefoperazone-sulbactam, cefepime, ceftriaxone, co-trimoxazole, tigecycline, aztreonam, minocycline, netilmicin, and colistin) and was intermediate susceptible to ciprofloxacin, piperacillin-tazobactam, gentamicin, meropenem, and amikacin, while it was resistant to none of the antimicrobials tested. The string test is a preliminary test for HMV, in which a string with a length of ≥5 mm extends upon touching of a colony of the test strain using an applicator and pulling away (7). P34 produced a string with a length of >65 mm and was considered string test positive (Fig. 1A). However, while the string test is simple to perform, it is not quantitative (5). Therefore, we performed a sedimentation assay by centrifuging suspensions of P34 along with two reference strains (ATCC BAA-2146 and ATCC 13883) and another clinical isolate from blood, BC21, at low speed (8). P34 did not sediment as well as any of the strains tested (Fig. 1B) and exhibited an optical density at 600 nm (OD_600_) supernatant/total ratio of ~0.8 (Fig. 1C), while other strains tested did not exceed 0.2. Given the source of P34, its susceptibility to most antibiotics, and its HMV nature, it was suspected to be of a hypervirulent lineage. Since most HMV and hypervirulent strains also overproduce CPS, we assessed CPS levels in P34 using the uronic acid assay (6). Surprisingly, P34 showed similar or lower levels of CPS, compared with other non-HMV strains tested in this study (Fig. 1D). This observation adds support to the growing notion in the field that HMV may be a distinct phenotype that does not require overproduction of CPS. However, CPS overproduction and HMV are important for invasive infection and enable immune evasion in hypervirulent K. pneumoniae (1). Therefore, we tested whether HMV of P34 offered protection from antimicrobial activities of human serum (H4522-100ML; Sigma) using a protocol described earlier, with modifications (9). P34 had a survival ratio (number of viable cells per milliliter after exposure to serum for 2 h divided by the number of viable cells per milliliter after exposure to phosphate-buffered saline [PBS] for 2 h) of 0.01 (Fig. 1E). A survival ratio of <1 indicates serum sensitivity, while a ratio of >1 indicates resistance to the antimicrobial activity of serum. However, no antimicrobial effect was observable when a similar suspension of P34 was treated with the same volume of heat-inactivated human serum (Fig. 1E). This finding suggested that P34 may be killed by complement-mediated lysis. However, we do not rule out killing by other heat-labile, antimicrobial components of human serum (10). Nevertheless, it was clear that, in the case of P34, HMV was unable to protect the cells from serum-mediated killing. This observation is consistent with the current understanding in the field that resistance to serum is driven primarily by CPS production, rather than HMV (11). Among other strains tested, which produced lower, similar, or higher levels of CPS but were non-HMV, ATCC 13883 had a survival ratio of 0.24 and tested sensitive to serum, while BC21 and ATCC BAA-2146 had survival ratios of 16.44 and 12.21, respectively, indicating serum resistance.

In order to better explain its unusual properties and to identify genetic determinants of the HMV phenotype in P34, we sequenced the genome of P34 on an Oxford Nanopore Technologies MinION device using an R9.4.1 flow cell. The data generated (654 Mb, with coverage of ~119×) were base called (Guppy v4.2.2; Oxford Nanopore Technologies) and assembled (Flye assembler v2.8) (12), producing a single circular genome (~5.13 Mb) with no detected plasmids. The assembly was then polished (medaka v1.3.4; Oxford Nanopore Technologies) and submitted to GenBank. P34 belonged to an unknown sequence type (ST), with the closest matches being ST55, ST1392, ST1393, and ST774. The capsular type was predicted to be KL131, while the O-antigen type was predicted to be O4. The genomic characteristics and virulence properties of P34 and a few other strains for comparison are presented in Table 1. The P34 resistome (13) harbored antibiotic resistance determinants such as *oqxA*, *oqxB* (trimethoprim, ciprofloxacin, nalidixic acid, chloramphenicol, and quaternary ammonium compounds), *fosA* (fosfomycin), and *bla*_SHV-172_ (β-lactams) and mutations in genes that are known to confer antimicrobial resistance such as *acrR* (fluoroquinolones), *ompK* (carbapenems and cephalosporins), and *ramR* (tigecycline), which might have contributed to its testing intermediate to ciprofloxacin, nalidixic acid, piperacillin-tazobactam, and meropenem. Since the *rmp* loci are known to be carried on ICEKp elements and plasmids, we ran BLASTn homology searches for ICEKp elements known in K. pneumoniae (14) and a PlasmidFinder 2.0 (15) search with the P34 genome as query. We were not able to find any significant homology to ICEKp elements or any significant hits to plasmids of *Enterobacteriales*. Moreover, key determinants of hypervirulence (Table 1) were also absent from the P34 genome. Surprisingly, the genome annotation pipelines RAST (16) and Prokka (17) also failed to detect the HMV-associated genes *rmpA*/*rmpA2*, *rmpD*, and *rmpC* in the P34 genome. We also ran BLASTn and tBLASTx searches with *rmpA* (locus tag VK055_RS25810, from K. pneumoniae ATCC 43816 KPPR1), *rmpA2* (locus tag LV136, from K. pneumoniae CG43 plasmid pLVPK), *rmpC* (locus tag VK055_RS25815, from K. pneumoniae ATCC 43816 KPPR1), and *rmpD* (locus tag VK055_RS28325, from K. pneumoniae ATCC 43816 KPPR1) as queries against the P34 genome but found no significant hits, although we could detect homologs of capsule synthesis regulators described earlier (18), such as Fur, RcsAB, KvrA, KvrB, CRP, H-NS, and IscR, in a BLASTn search. Therefore, it appears that the HMV phenotype in P34 is encoded by determinants other than the *rmpA*, *rmpD*, and *rmpC* loci. Earlier studies from India and abroad also reported HMV strains of K. pneumoniae and Klebsiella quasipneumoniae, which do not harbor *rmpA* (19–24). Given that HMV is a virulence factor known to prevent adherence and internalization of K. pneumoniae by macrophages *in vitro* (5), it is important to study the genetic basis of HMV in isolates, like P34, that do not harbor the *rmp* loci. Furthermore, the potential spread of these unknown HMV determinants via horizontal gene transfer to other non-HMV K. pneumoniae strains and the possible acquisition of virulence and drug resistance plasmids by P34-like strains are viable threats to public health that need further investigation.

**TABLE 1:** Genome and virulome characteristics of Klebsiella pneumoniae P34 in comparison with other strains

Category and feature	Data for strain:					Tool and reference
	ATCC 13883	ATCC bAA-2146	BC21 (unpublished)	P34	ATCC 43816	
Genome						
Size (bp)	5,548,441	5,781,501	5,980,523	5,135,526	5,362,708	
GC content (%)	57	57	56.7	57.5	57.4	
No. of coding sequences	5,142	5,510	6,155	5,131	4,924	Prokka (17)
No. of tRNA genes	83	85	88	86	85	
No. of rRNA genes	25	25	25	25	25	
No. of miscellaneous RNA genes	137	152	163	126	124	
No. of transfer-messenger RNA genes	1	1	1	1	1	
ST	3	11	Closest to 15 with 3 locus variants	Unknown; closest matches: 55, 1392, 1393, 774	493	MLST v2.0 (25)
Virulome profile						
Capsule type	KL3 (99.6%), mannose	KL74 (98.88%), mannose	KL2 (99.47%), mannose	KL131 (99.74% identity), rhamnose	KL2 (99.72%), mannose	Kleborate (26)
Capsule overproduction						
* rmpA*	+	−	−	−	+	BLASTn (27)
* rmpA2*	−	−	−	−	−	
* rmpC*	+	−	−	−	+	
Lipopolysaccharides						
O-antigen	O1v1 (98.28% identity)	O3b (91.71%)	O1v1 (98.47%)	O4 (99.64%)	O1v1 (99.81%)	Kleborate (26)
* rfb* locus	+	+	+	−	+	VF Analyzer (28)
HMV						
String test	−	−	−	+	+	
* rmpD*	−	−	−	−	+	BLASTn (27)
Adhesins						VF Analyzer (28)
Type 3 fimbriae	+	+	+	+	+	
Type I fimbriae	+	+	+	+	+	
Type IV pili (*pilW*)	+	−	−	+	−	
Siderophores						
Aerobactin biosynthesis	−	−	−	−	−	
Aerobactin transport	+	+	+	+	+	
Enterobactin biosynthesis and transport	+	+	+	+	+	
Salmochelin biosynthesis	−	−	−	−	+	
Salmochelin transport	+	+	+	+	+	
Yersiniabactin biosynthesis	+	−	+	−	+	
Yersiniabactin transport	+	−	+	−	+	
Nutritional factors						
Allantoin utilization	−	−	−	−	−	
Other virulence factors						
Colibactin biosynthesis	+	−	−	−	−	
Tellurite resistance (*terB*)	+	−	+	+	+	BLASTn (27)
* peg-344*	+	−	−	−	+	
* peg-589*	−	−	−	−	−	

### Data availability.

The P34 genome assembly was submitted to NCBI with accession number CP076526.
